# A reduction in growth rate of *Pseudomonas putida *KT2442 counteracts productivity advances in medium-chain-length polyhydroxyalkanoate production from gluconate

**DOI:** 10.1186/1475-2859-10-25

**Published:** 2011-04-22

**Authors:** Stéphanie Follonier, Sven Panke, Manfred Zinn

**Affiliations:** 1 Laboratory for Biomaterials, Swiss Federal Laboratories for Materials Science and Technology (Empa), Lerchenfeldstrasse 5, 9000 St. Gallen, Switzerland; 2 Bioprocess Laboratory, Department of Biosystems Science and Engineering (D-BSSE), ETH Zurich, Mattenstrasse 26, 4058 Basel, Switzerland

## Abstract

**Background:**

The substitution of plastics based on fossil raw material by biodegradable plastics produced from renewable resources is of crucial importance in a context of oil scarcity and overflowing plastic landfills. One of the most promising organisms for the manufacturing of medium-chain-length polyhydroxyalkanoates (mcl-PHA) is *Pseudomonas putida *KT2440 which can accumulate large amounts of polymer from cheap substrates such as glucose. Current research focuses on enhancing the strain production capacity and synthesizing polymers with novel material properties. Many of the corresponding protocols for strain engineering rely on the rifampicin-resistant variant, *P. putida *KT2442. However, it remains unclear whether these two strains can be treated as equivalent in terms of mcl-PHA production, as the underlying antibiotic resistance mechanism involves a modification in the RNA polymerase and thus has ample potential for interfering with global transcription.

**Results:**

To assess PHA production in *P. putida *KT2440 and KT2442, we characterized the growth and PHA accumulation on three categories of substrate: PHA-related (octanoate), PHA-unrelated (gluconate) and poor PHA substrate (citrate). The strains showed clear differences of growth rate on gluconate and citrate (reduction for KT2442 > 3-fold and > 1.5-fold, respectively) but not on octanoate. In addition, *P*. *putida *KT2442 PHA-free biomass significantly decreased after nitrogen depletion on gluconate. In an attempt to narrow down the range of possible reasons for this different behavior, the uptake of gluconate and extracellular release of the oxidized product 2-ketogluconate were measured. The results suggested that the reason has to be an inefficient transport or metabolization of 2-ketogluconate while an alteration of gluconate uptake and conversion to 2-ketogluconate could be excluded.

**Conclusions:**

The study illustrates that the recruitment of a pleiotropic mutation, whose effects might reach deep into physiological regulation, effectively makes *P. putida *KT2440 and KT2442 two different strains in terms of mcl-PHA production. The differences include the onset of mcl-PHA production (nitrogen limitation) and the resulting strain performance (growth rate). It remains difficult to predict a prioriwhere such major changes might occur, as illustrated by the comparable behavior on octanoate. Consequently, experimental data on mcl-PHA production acquired for *P. putida *KT2442 cannot always be extrapolated to KT2440 and vice versa, which potentially reduces the body of available knowledge for each of these two model strains for mcl-PHA production substantially.

## Background

Medium-chain-length polyhydroxyalkanoates (mcl-PHAs) are polyesters which combine the features of biodegradability, biocompatibility, and production from renewable carbon sources and as such constitute a promising alternative to petrol-based plastics [1]. Mcl-PHAs are formed of monomers with 6 to 14 carbon atoms, in contrast to the short-chain-length polyhydroxyalkanoates (scl-PHAs) that contain 3 to 5 carbons per monomer. They are synthetized by Pseudomonads, principally under carbon excess conditions [2]. *Pseudomonas putida *KT2440 and KT2442 belong to the best-known producers of mcl-PHA. They accumulate polymer from both PHA-related carbon sources (e. g. fatty acids) via β-oxidation and from PHA-unrelated carbon sources (e. g. sugars) via *de novo *fatty acid synthesis [2,3]. *P. putida *KT2440, whose genome was sequenced a decade ago [4], originates from the toluene-degrading bacterium *P. putida *mt-2 isolated in Japan in 1960 by Hosakawa [5]. *P. putida *KT2442 is a spontaneous rifampicin resistant mutant of *P. putida *KT2440 [6,7] proposed to have a similar expression profile as *P. putida *KT2440 [8]. Currently, much effort is spent on engineering these strains in order to increase their accumulation capacity, for instance by deletion of depolymerases [9], and in modifying the pathways involved in PHA synthesis so as to get polymers with modified compositions and improved material properties [10-13]. Knockout mutants are preferentially generated from the KT2442 strain as advantage can be taken from the rifampicin resistance to simplify the procedure. The mode of action of rifampicin consists of inhibiting bacterial growth by binding to the β-subunit of RNA polymerase and stopping mRNA elongation [14]. Rifampicin-resistant mutants have an altered β-subunit of RNA polymerase [15,16] and therefore their transcription profiles and physiology can be significantly affected.

Since *P. putida *KT2440 and KT2442 are mostly used for the production of mcl-PHA, we compared their performances on octanoate, a PHA-related carbon source, and on gluconate, a PHA-unrelated source. Citrate was used as control since it is a poor PHA-precursor (shown *a posteriori*, see *Methods *and *Results*). The two key parameters influencing PHA productivity (in g L^-1 ^h^-1^) and as a result the production costs are the cell growth rate (in h^-1^) and the maximum PHA content (in wt %). The latter is especially important because a high PHA content additionally simplifies the down-stream process and thus decreases costs. Therefore, we performed shake flask experiments to determine these factors for the three growth substrates. It should be noted that the values reported here are not representative of optimized processes but qualitatively express if one strain shows better performances than the other one or not. The growth of *P. putida *KT2440 and KT2442 on gluconate was studied in more detail in a well-controlled bioreactor setting after discovering major physiological differences between the two strains.

In this work, we demonstrated that *P. putida *KT2440 and KT2442 produced mcl-PHA from the fatty acid octanoate with similar efficiency but that *P. putida *KT2442 had a strongly reduced productivity on gluconate because of a more than 3-fold smaller growth rate.

## Results

### *P. putida *KT2442 exhibits reduced specific growth rate and production of mcl-PHA on gluconate compared to its parent KT2440

**Figure 1:**
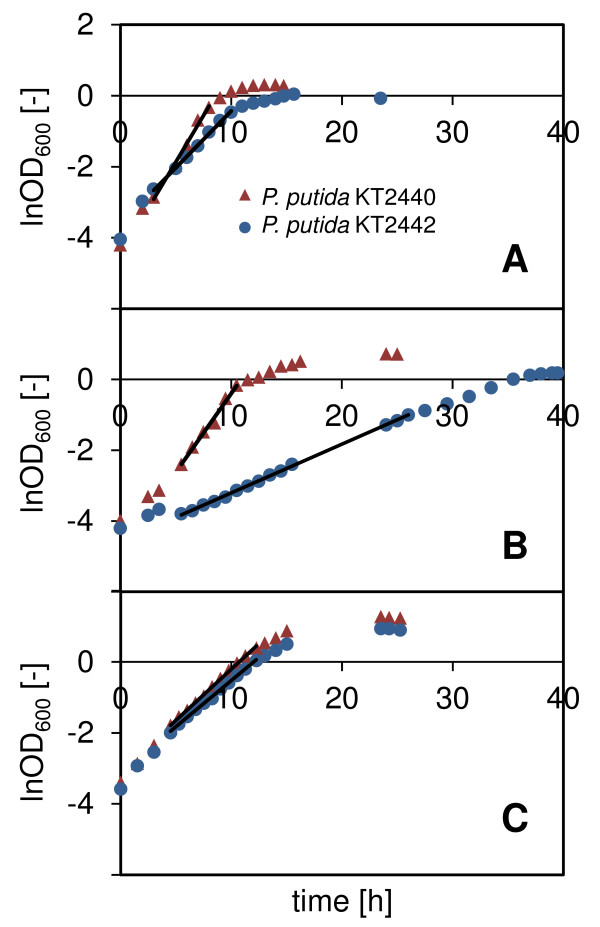
**Growth of *P. putida *KT2440 and KT2442 on citrate, gluconate, and octanoate**. Example of the growth curves obtained in shake flasks for *P. putida *KT2440 and KT2442 cultivated at 30°C in mineral medium supplemented with (A) trisodium citrate dihydrate, (B) sodium gluconate, and (C) sodium octanoate. The straight lines indicate the regimes of exponential growth that were considered for calculation of the maximum specific growth rate.

**Figure 2:**
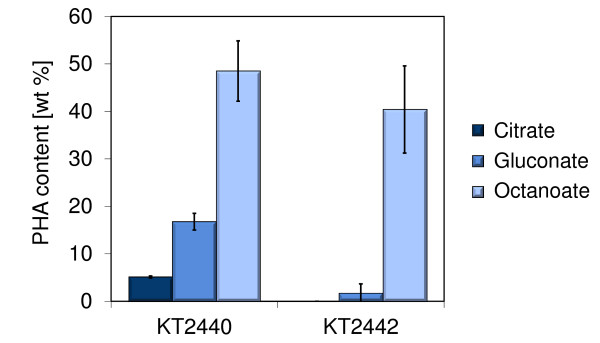
**PHA content in *P. putida *KT2440 and KT2442 cultivated on citrate, gluconate, and octanoate**. The PHA contents correspond to samples taken at the end of the growth experiments (last OD_600 _measurement in Figure 1). The relative weight amount of PHA in freeze-dried biomass was determined by gas chromatography. The error bars represent the standard deviation of PHA content from 2-4 independent experiments.

*P. putida *KT2440 and KT2442 were cultivated at 30°C in shake flasks containing mineral medium and either octanoate, gluconate, or citrate as carbon source. The three media had a C/N ratio of 15 g g^-1 ^and the same carbon concentration, which was low in comparison to industrial production processes so as to stay below the growth inhibitory level of octanoate (about 15 mM, data not shown) without having to implement complex feeding schemes. *P. putida *KT2440 and KT2442 exhibited a similar maximum specific growth rate on octanoate (0.26 ± 0.01 h^-1 ^and 0.29 ± 0.01 h^-1^, respectively) and accumulated mcl-PHA to similar extents (48 ± 2 and 40 ± 10 wt %, respectively) (Figure 1 and 2). In contrast, the two strains behaved differently when grown on PHA-unrelated carbon sources. The maximum specific growth rate of *P*. *putida *KT2442 was substantially smaller than that of *P. putida *KT2440 on citrate (0.34 ± 0.02 h^-1 ^and 0.54 ± 0.03 h^-1^, respectively), and the difference was even more pronounced on gluconate (0.13 ± 0.01 h^-1 ^and 0.43 ± 0.02 h^-1^, respectively). This suggests that the uptake and/or metabolism of citrate and gluconate but not that of fatty acids is altered in *P. putida *KT2442. *P. putida *KT2442 also had a decreased maximum specific growth rate (> 2-fold) on glucose (data not shown). In addition to the impaired growth, the production of mcl-PHA by *P. putida *KT2442 was much lower than that of *P. putida *KT2440 when gluconate was used as substrate (Figure 2). Only a negligible amount of polymer could be detected in this strain (1.7 ± 2.1 wt %) whereas *P. putida *KT2440 accumulated 16.8 ± 0.6 wt %. This was unexpected, since *P. putida *KT2442 has been reported several times to produce PHA from unrelated sources [3,17]. We thus investigated this further with cultivations in bioreactor which have a better controlled environment and enable a precise monitoring of the process due to a larger volume for sampling. Finally, it should also be noted that the 5 wt % PHA detected for *P. putida *KT2440 cultivated on citrate may have arisen from contamination with cell membrane components and not be actual polymer (see the section *Methods*).

### *P. putida *KT2442 can produce mcl-PHA from gluconate when the C/N ratio is increased

**Figure 3:**
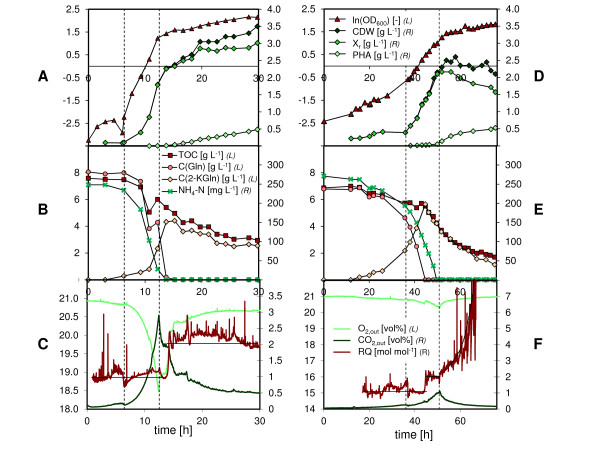
**Fermentation of *P. putida *KT2440 and KT2442 on gluconate**. The process data for the fermentation of *P. putida *KT2440 and KT2442 on sodium gluconate (21.2 g L^-1^) are depicted in the panels A-C and D-F, respectively. Biomass growth is represented in panels A and D with ln(OD_600_) and with total biomass (CDW), PHA-free biomass (X_r_), and PHA. The residual concentrations of organic carbon (TOC), carbon from gluconate (C(Gln)), and carbon from 2-ketogluconate (C(2-KGln)) can be read in panels B and E. These three values are expressed in g C L^-1 ^to facilitate their comparison. In the same panels is also shown the residual concentration of ammonium nitrogen (NH_4_-N). In panels C and F are displayed the off-gas concentrations of oxygen and carbon dioxide, and the respiratory quotient (RQ). The two dashed lines indicate the exponential growth phases. Whether the values must be read on the left (L) or on the right (R) axes is written in the labels.

In order to study in more detail the differences of physiology upon growth on gluconate between *P. putida *KT2440 and KT442, batch fermentations were performed in a 16 L bioreactor (V_w _= 11 L) (Figure 3). Firstly, *P. putida *KT2442 was cultivated on gluconate with the same medium composition as for the shake flask experiments (data not shown). This experiment revealed that under these conditions (C/N ratio = 15 g g^-1^) and unlike for *P. putida *KT2440 nitrogen and carbon depletion occurred simultaneously. This would explain why no PHA was detected in KT2442 during the shake flask experiments since *P. putida *KT2440 requires nitrogen limitation for synthesizing polymer from gluconate but not from fatty acids [18]. In order to test this hypothesis the initial C/N ratio was increased to 22.5 g g^-1^, and the concentrations of both the carbon and the nitrogen sources were increased so as to have more biomass available for analyses. The slower growth rate of *P. putida *KT2442 on gluconate was confirmed at this larger scale of cultivation, its maximum specific growth rate being more than 4 times smaller than the one of *P. putida *KT2440 (0.13 ± 0.01 h^-1 ^and 0.56 ± 0.03 h^-1^, respectively). In addition, *P. putida *KT2442 was able to accumulate polymer to a similar extent as *P. putida *KT2440 now that the C/N ratio was increased by 7.5 g g^-1 ^and that carbon was available for PHA synthesis at the time of nitrogen depletion (Figure 3).

### *P. putida *KT2442 PHA-free biomass decreases during nitrogen limiting growth on gluconate

Apart from the maximum specific growth rate, the growth profiles of *P. putida *KT2440 and KT2442 were rather similar during the cultivation on gluconate in the bioreactor (Figure 3). After a lag phase during which the cells adapted their metabolism from citrate (preculture) to gluconate the cells entered the exponential growth phase. This phase was characterized by a fast consumption of gluconate and the release of 2-ketogluconate as overflow metabolite. Gluconate depletion occurred during the exponential growth phase for *P. putida *KT2442 and was followed by the consumption of the previously released 2-ketogluconate. The transition from gluconate to the more oxidized 2-ketogluconate was marked by a sharp increase of the respiratory quotient (RQ) from ~1 to ~2 mol mol^-1 ^for both *P. putida *KT2440 and KT2442 (Figure 3C and 3F). However, no change of growth rate was observed, which indicates that no major cellular adaptation was required (Figure 3D and 3E). These observations are consistent with the ones made by Latrach Tlemçani *et al*. who cultivated *P. putida *mt-2 on glucose and measured a change of RQ and specific growth rate from 0.96 to 1.43 mol mol^-1 ^and from 0.32 to 0.29 h^-1^, respectively, between the growth phase on gluconate and the growth phase on 2-ketogluconate [19]. The exponential phase ended for both strains with nitrogen starvation and subsequently the PHA accumulation phase started. The transition between the exponential and the accumulation phase is clearly indicated by the peaks of oxygen and carbon dioxide concentrations in the off-gas (Figure 3C and 3F). The production of PHA was similar in both *P. putida *KT2440 and KT2442 that accumulated PHA with a specific rate of 0.012 and 0.008 g g^-1 ^h^-1^, respectively (Table 1). Noticeably, the total cell dry weight and the PHA-free biomass decreased during the accumulation phase for *P. putida *KT2442, whereas it first slightly increased during 7.5 h and then remained stable for *P. putida *KT2440. Also, the progressive increase of RQ following nitrogen depletion (Figure 3F) suggested that KT2442 was impaired in its metabolism.

**Table 1:** Specific uptake and production rates of *P. putida *KT2440 and KT2442 cultivated in bioreactor on gluconate (21.2 g L^-1^)

	KT2440	KT2442
*Exponential phase*		
q_C(Gln) _[g g h^-1^]	-2.0	-0.7^a^/0.0^b^
q_C(2-KGln) _[g g h^-1^]	+1.0	+0.5^a^/-0.2^b^
q_C* _[g g h^-1^]	-1.0	-0.2^a^/-0.2^b^
q_N _[g g h^-1^]	-0.062	-0.015
*Accumulation phase*		
q_C(Gln) _[g g h^-1^]	-1.3	0.0
q_C(2-KGln) _[g g h^-1^]	-0.07	-0.07
q_PHA_[g g h^-1^]	0.012	0.008

a: before gluconate depletion

b: after gluconate depletion

### The conversion of gluconate into 2-ketogluconate is not affected in *P. putida *KT2442

In order to further investigate the nutrient uptake in *P. putida *KT2440 and KT2442, their specific carbon and nitrogen uptake rates were compared using the data from the batch experiments on gluconate in bioreactors. We defined the specific carbon consumption rate q_C* _as the specific rate at which carbon was utilized by the cells for respiration and production of biomass, and assumed that there was no accumulation of the substrate gluconate and of its metabolite 2-ketogluconate in the periplasm. Consequently, q_C* _could be calculated as the difference between the specific uptake rate of gluconate and the specific production rate of 2-ketogluconate (in g C). This expression could only be used until gluconate depletion. Afterwards, q_C* _was equivalent to the specific uptake rate of 2-ketogluconate (see the section *Methods *for the detailed calculations). Both the specific carbon and nitrogen uptake rates were 4-5 times lower for *P. putida *KT2442 than for *P. putida *KT2440 during the exponential phase (q_C* _= - 1.0 and - 0.2 g g^-1 ^h^-1^, q_N _= - 0.062 and - 0.015 g g^-1 ^h^-1 ^for *P. putida *KT2440 and KT2442, respectively) (Table 1). This reduction fits well with the > 4-fold difference of growth rate between the strains, but whether the decreased uptake rates in *P. putida *KT2442 are the cause or the result of the slow growth remains unclear. In addition, the specific production rate of 2-ketogluconate of *P. putida *KT2442 was only half of the rate of *P. putida *KT2440 (q_C(2-KGln) _= + 0.5 and + 1.0 g g^-1 ^h^-1^, respectively). The extent of gluconate converted into extracellular 2-ketogluconate was calculated from the rates q_C(Gln) _and q_C(2-KGln) _for *P. putida *KT2440 and KT2442. It was equal to 50% and >70%, respectively, which implies that the oxidation of gluconate was fully functional in *P. putida *KT2442.

In order to determine whether the biomass production from gluconate was affected in *P. putida *KT2442, the growth yields for PHA-free biomass on carbon and nitrogen (Y_Xr/C _and Y_Xr/N_) were calculated for *P. putida *KT2440 and KT2442, both at the end of the exponential phase and at the end of the PHA accumulation phase (Table 2). At the end of the exponential phase, the two strains exhibited the same Y_Xr/N _(~8 g g^-1^). In contrast, *P. putida *KT2442 had a smaller Y_Xr/C _(0.82 vs 1.23 g g^-1 ^for *P. putida *KT2440) which explains why nitrogen limitation occurred comparatively earlier for *P. putida *KT2440 during batch cultures. At the end of the accumulation phase, both Y_Xr/C _and Y_Xr/N _were significantly smaller for *P. putida *KT2442 (Table 2) which was a consequence of the decrease of rest biomass after nitrogen depletion for this strain (Figure 3). The PHA yield for carbon (Y_PHA/C_) at the end of the cultivation was however the same for both strains (0.09 g PHA g^-1 ^C) reflecting the fact that no PHA was degraded.

**Table 2:** Growth and PHA yields of *P. putida *KT2440 and KT2442 cultivated in bioreactor on gluconate (21.2 g L^-1^)

	KT2440	KT2442
*End exp. phase*		
Y_Xr/C _[g g^-1^]	1.23	0.82
Y_Xr/N _[g g^-1^]	8.0	7.8
*End acc. phase*		
Y_Xr/C _[g g^-1^]	0.53	0.28
Y_Xr/N _[g g^-1^]	11.7	5.8
Y_PHA/C _[g g^-1^]	0.09	0.09

## Discussion

### How close are *P. putida *KT2440 and KT2442?

*P. putida *KT2440 and *P. putida *KT2442 have been extensively studied for the last 30 years, not only because of their interesting ability to accumulate mcl-PHA, but also as model organisms for laboratory studies and applications in bioremediation and biocatalysis [20,21]. *P. putida *KT2440 and *P. putida *KT2442 are supposedly identical except regarding the rifampicin resistance. However, we observed different phenotypes between the two strains cultivated on gluconate: *P. putida *KT2442 displayed a reduced growth rate along with a lower growth yield for carbon and difficulties to cope with nitrogen limitation.

Rifampicin activity consists of inhibiting the bacterial DNA-dependent RNA polymerase by binding to it and stopping mRNA elongation [14]. Rifampicin-resistant mutants produce RNA polymerases that have a slightly different β-subunit structure preventing the binding of rifampicin [16]. Jatsenko *et al*. also showed recently that out of 167 rifampicin resistant mutants generated from *P. putida *PaW85 (*P. putida *mt-2 derivative cured of the TOL plasmid), all of them harbored the mutation of interest in the cluster I of *rpoB *gene [15]. Because of the essential role of the RNA polymerase in gene transcription even slight modifications of its structure can have important and pleiotropic effects on the cell physiology. In particular, the regulatory nucleotide ppGpp was shown to bind at the interface between the β and β' subunits of *E. coli *RNA polymerase at 27 Å from the rifampicin binding site [22-25]. Therefore, modifications of RNA polymerase structure, even minor, could alter the binding of ppGpp with various consequences on the cell physiology. Indeed, ppGpp is a global transcription regulator mostly known for inhibiting growth and protein synthesis upon amino acid starvation but also involved in the regulation of many other functions [24,26]. Besides, since *P. putida *KT2442 is a spontaneous mutant of *P. putida *KT2440 and not the result of a targeted procedure [6], the strain could harbor other mutations that may be made responsible for its poorer fitness. The determination of this (these) mutation(s) would require careful resequencing of KT2442. Nevertheless, a first step would be to determine whether the rifampicin resistance mutation is involved by reintroducing the wild-type *rpoB *gene from *P. putida *KT2440 in KT2442.

### Gluconate transport and metabolism

**Figure 4:**
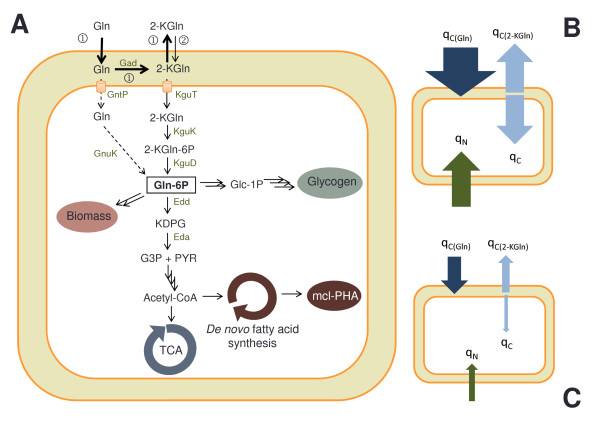
**Gluconate metabolism in *P. putida *strains KT2440 and KT2442**. A. The route before gluconate depletion is indicated by the numbers "1" and the route after gluconate depletion one with "2". The dotted arrows describe the direct transport of gluconate into the cytoplasm which is possible but of minor importance. This figure was adapted from Daddaoua *et al*. [31]. Abbreviations: Eda = 2-keto-3-deoxygluconate aldolase, Edd = phosphogluconate dehydratase, Gad = gluconate dehydrogenase, Glc-1P = glucose-1-phosphate, Gln = gluconate, Gln-6P = 6-phosphogluconate, GntP = gluconate permease, Gnuk = gluconokinase, G3P = glyceraldhyde 3-phosphate, KDPG = 2-keto-3-deoxy-6-phosphogluconate, 2-KGln = 2-ketogluconate, 2-KGln-6P = 2-keto-6-phosphogluconate, KguD = 2-ketogluconate reductase, KguK = 2-ketogluconate kinase, KguT = 2-ketogluconate transporter, PYR = pyruvate, TCA = tricarboxylic acid cycle. B. Specific uptake rate of gluconate (q_C(Gln)_), specific production rate of 2-ketogluconate (q_C(2-KGln)_), carbon specific uptake rate (q_C*_) and nitrogen specific uptake rate (q_N_) for *P. putida *KT2440 growing exponentially on gluconate in bioreactor. The width of the arrows expresses the actual values. C. Same as B but for *P. putida *KT2442.

The difference of growth rate observed between *P. putida *KT2440 and KT2442 on gluconate can reside in the transport of gluconate, in its metabolism, or in their regulation. The general model describing the transport and metabolism of gluconate in *P. putida *is depicted in Figure 4. First, gluconate crosses the outer membrane by facilitated diffusion mostly through the specific porin OprD, which is also utilized by basic amino acids [27]. The role of this porin is only important at low substrate concentrations which would correspond to the end of the exponential phase in our experiments. Indeed, similar growth rates were observed between the wild-type *P*. *aeruginosa *strain and its OprD mutant on gluconate 10 mM whereas the OprD mutant exhibited a 3-fold reduced growth rate on gluconate 1 mM [27]. Therefore, a lack of OprD porins cannot explain the reduced growth rate of *P. putida *KT2442 during the exponential phase. Once in the periplasm, gluconate can pass through the cytoplasmic membrane via the active transporter GntP. The transport of gluconate and its subsequent phosphorylation in the cytoplasm in under the control of GnuR repressor [28]. Nevertheless, this route is not the preferred one, gluconate being preferentially converted into 2-ketogluconate by Gad enzymes bound to the periplasmic side of the cytoplasmic membrane [29,30]. It was observed in this work that 50% of the gluconate taken up during the exponential growth phase was converted into 2-ketogluconate by *P. putida *KT2440 and secreted (q_C(Gln) _= - 2.0 g g^-1 ^h^-1^, q_C(2-KGln) _= + 1.0 g g^-1 ^h^-1^; Table 1, Figure 4B) whereas >70% of the produced 2-ketogluconate was released in the extracellular fraction in case of *P. putida *KT2442 (q_C(Gln) _= - 0.7 g g^-1 ^h^-1^, q_C(2-KGln) _= + 0.5 g g^-1 ^h^-1^;Table 1, Figure 4C). The conversion of gluconate into 2-ketogluconate was thus working efficiently in *P. putida *KT2442. The molecules of 2-ketogluconate that are not secreted into the medium are actively transported in the cytoplasm by KguT proteins (putative transporter gene PP_3377 [29]). There, the molecules are phosphorylated and further metabolized for energy production via the Entner-Doudoroff pathway and for biomass production. Interestingly, the genes involved in the periplasmic conversion of gluconate to 2-ketogluconate and the genes responsible for the transport and cytoplasmic conversion of 2-ketogluconate to 6-phosphogluconate are clustered in two independent operons located next to each other. These two operons are under the control of a PtxS regulator, which specifically recognizes 2-ketogluconate [31]. If growth is stoichiometrically limited, for instance by nitrogen, the excess of carbon can be accumulated as a storage compound such as glycogen or mcl-PHA. The production of glycogen was however negligible under the growth conditions tested (< 4 wt %, data not shown) and most of the excess carbon was directed towards synthesis of mcl-PHA. An inefficient transport of 2-ketogluconate through the cytoplasmic membrane or an impaired step in the further metabolization would therefore be reasonable explanations for the reduced growth rate of *P. putida *KT2442.

### Energy metabolism, nitrogen transport, and growth under nitrogen limitation

*P. putida *KT2440 and KT2442 grew with the same maximum specific growth rate on octanoate, indicating that the main energy production pathway (tricarboxylic acid cycle) worked properly. The slow growth rate observed for *P. putida *KT2442 on gluconate is therefore substrate-specific. However, *P. putida *KT2442 grew more slowly than *P. putida *KT2440 on citrate (μ_max _= 0.34 ± 0.02 h^-1 ^and 0.54 ± 0.03 h^-1^, respectively). This could be the result of a problem with the expression of a citrate transporter or activator of citrate transport. An alternative explanation would be that the tricarboxylic acid cycle was only slightly slowed down in *P. putida *KT2442 so that it became growth limiting only when the cells were growing fast (e. g. on citrate) but not when they were growing more slowly (e. g. on octanoate).

When ammonium is present at high external concentrations as during the exponential phase, it enters the cytoplasm via unspecific diffusion of NH_3 _[32] and does not require specific transporters. Therefore, the low specific uptake rate of nitrogen for *P*. *putida *KT2442 must be the consequence and not the cause of its slow growth rate. This conclusion is also supported by the fact that *P. putida *KT2442 and KT2440 had identical growth rates on octanoate.

*P. putida *KT2440 and KT2442 reacted differently with respect to nitrogen starvation. While the PHA-free biomass continued to increase a little and then remained constant for *P. putida *KT2440, it quickly decreased for *P. putida *KT2442 (Figure 4A and 4D). Also, the respiratory quotient of the latter strain progressively and significantly increased, whereas it remained stable for *P. putida *KT2440. This indicates a change of metabolism occurring for *P. putida *KT2442 as a result to nitrogen starvation. Although the gene expression in response to nitrogen limitation has been studied for *P. putida *KT2440 and KT2442 by Hervas *et al*. [8], it was not possible to formulate a reasonable hypothesis as to what caused this different behavior.

### Nitrogen limitation is required for the accumulation of mcl-PHA from gluconate in *P. putida *KT2440 and KT2442

The data presented herein showed that nitrogen limitation was required to activate the production of mcl-PHA from gluconate in both *P. putida *KT2440 and KT2442. In contrast, we had observed previously that *P. putida *KT2440 cultivated on octanoate accumulated significant amounts of mcl-PHA even before nitrogen depletion (data not shown). Also, Sun *et al*. were able to produce more than 70 wt % of mcl-PHA from nonanoic acid in *P. putida *KT2440 without nitrogen limitation. Therefore, the requirement of a nutrient limitation to produce mcl-PHA seems to be substrate-dependent. As mentioned above, the synthesis of mcl-PHA by *P. putida *involves two different pathways depending on the precursor. Unrelated carbon sources such as gluconate are converted into polymer via *de novo *fatty acid synthesis and the intervention of the 3-hydroxy-acyl carrier protein (ACP)-CoA transacylase PhaG [33], whereas alkanes and fatty acids are channeled through the β-oxidation pathway [34]. The origin for the need or not of nitrogen limitation may thus be linked to enzymes belonging to these pathways. Indeed, the enzyme PhaG was shown to be overexpressed under nitrogen starvation in *P. putida *KT2440 and KT2442 [8]. However, other control systems may be involved as well.

## Conclusions

This work shows substantial differences of physiology between the two closely related *P. putida *KT2440 and KT2442 upon growth on gluconate. A strong reduction of specific growth rate was observed for *P. putida *KT2442 as well as a smaller growth yield on carbon and difficulties to cope with nitrogen starvation. *P. putida *KT2442 is often preferred over *P. putida *KT2440 to generate metabolically engineered organisms with enhanced production of mcl-PHA because the procedure is simplified by the rifampicin resistance [17,35]. However, the productivity of mcl-PHA - which is correlated to its cost - depends not only on the polymer content in the cells but also on their growth rate. Thus, the benefits of an increase in polymer accumulation from carbohydrates by metabolic engineering would be counteracted by a slow growth rate if *P. putida *KT2442 was used as host instead of *P. putida *KT2440.

## Methods

### Strains and media

The strains used in this study are *P. putida *KT2440 (own lab stock) and its rifampicin resistant derivative *P. putida *KT2442 [6] which was kindly provided by M. A. Prieto (CIB, Madrid, Spain). The slow growth rate of *P. putida *KT2442 on gluconate was verified with a strain from our stock that originates from the lab of B. Witholt (ETHZ, Zurich, Switzerland).

The mineral medium used for the flask experiments was a modified medium E2 [36] with a reduced concentration of nitrogen (0.064 g N L^-1^). It was supplemented with either sodium octanoate (1.66 g L^-1^), sodium gluconate (2.91 g L^-1^) or trisodium citrate dihydrate (3.92 g L^-1^) as carbon source. Thus, the three media had the same carbon concentration (0.96 g C L^-1^) and the same C/N ratio (15 g g^-1^).

The medium for the batch cultivations in bioreactor had the following composition: 4.8 g L^-1 ^NaNH_4_PO_4_·4H_2_O (0.32 g N L^-1^), 21.2 g L^-1 ^sodium gluconate (7.2 g C L^-1^), 3.7 g L^-1 ^KH_2_PO_4_, 9.6 g L^-1 ^K_2_HPO_4_, 0.03 g L^-1 ^CaCl_2_·2H_2_O, 0.8 g L^-1 ^EDTANa·2H_2_O, 1 g L^-1 ^MgSO_4_·7H_2_O, 0.28 g L^-1 ^FeSO_4_·7H_2_O and 2 mL L^-1 ^of the following trace element solution: 12.22 g MnCl_2_·4H_2_O, 1.27 CoCl_2_·6H_2_O, 2.0 g CuCl_2_·2H_2_O, 7.5 g ZnSO_4_·7H_2_O, 0.5 g Na_2_MoO_4_·2H_2_O dissolved in 1 L HCl (1M). This medium had a C/N ratio of 22.5 g g^-1 ^and was more concentrated to achieve higher cell density.

### Shake flask experiments

Single colonies of the respective strains *P. putida *KT2440 and KT2442 were picked from a freshly streaked Luria-Bertani (LB) agar plate and grown overnight in LB medium at 30°C and 150 rpm. Three mL of these cultures were washed with mineral medium and used to inoculate 150 mL of each culture media (citrate, gluconate, and octanoate). The cultivation experiments were performed in 500 mL flasks with baffles shaken at 30°C and 150 rpm. The growth of each strain was studied in duplicates. Independent growth experiments were repeated in case of unexpected results; each time the first results were confirmed. At the end of the growth experiments, the biomass was collected for PHA analysis. The maximal specific growth rates and mcl-PHA contents were averaged from 2-4 values.

### Fermentations in bioreactor

The fermentations in bioreactor were performed in a 16 L bioreactor (L1523, Bioengineering, Wald, Switzerland) with a starting volume of 11 L. Inoculation was done with 250 mL of an exponentially growing culture in modified medium E [36]. Sterile polypropylene glycol was added to the culture to reduce foaming if necessary. The reactor was aerated with air (0.54 and 0.50 vvm for KT2440 and KT2442, respectively) and agitation varied from 300 to 700 rpm in order to avoid oxygen limitation. The temperature was set to 30°C and the pH kept between 6.85 and 6.95 by automated addition of KOH (4 M) and H_3_PO_4 _(4 M). The reactor was equipped with probes for pH and pO_2 _(Mettler Toledo, Greifensee, Switzerland). The off-gas concentrations of oxygen and carbon dioxide were monitored with a gas analyzer (BlueSens, Herten, Germany). The batch medium was autoclaved *in situ *without EDTANa·2H_2_O, MgSO_4_·7H_2_O, FeSO_4_·7H_2_O and gluconate that were sterile-filtered (0.22 μm pore size Millex-GP filter, Millipore, Billerica, USA) into the reactor after cooling.

### Analytical methods

*Biomass*. The optical density at 600 nm (OD_600_) of the cell culture was measured with a spectrophometer (Spectronic Genesys 6, Thermo Electron Corp., UK) and cell dry weight (CDW) concentrations were determined as described by Hartmann *et al*. [37]. *Residual concentrations of substrate*. The concentration of ammonium nitrogen (NH_4_-N) was assessed by spectrophotometry (Ammonium-Test Spectroquant 1.14752.0001, Merck, Darmstadt, Germany) and the total organic carbon (TOC) was analyzed with a TOC-Analyzer (model TOC-5050A, Shimadzu, Reinach, Switzerland). The concentrations of gluconate and 2-ketogluconate were determined by HPLC-MS (Agilent 1000 Series, Santa Clara, United States for the HPLC unit, and Bruker Daltonics esquire HCT, Bremen, Germany for the MS unit). The column used was a Gemini C18 250 mm × 4.60 mm and 5 μm particle size (Phenomenex, Torrance, United States) operated isocratically with 90% of solvent A (H_2_O and 0.1% HCOOH) and 10% of solvent B (CH_3_CH and 0.1% HCOOH) for 15 min with a flow rate of 0.5 mL min^-1^. Ionisation was performed by atmospheric pressure chemical ionisation (ACPI) in negative mode. Xylose was used as internal standard. *PHA content and composition*. The PHA production was characterized by gas chromatography (GC) after propylation of the monomers according to Furrer *et al*. [38]. PHA contents lower than 5 wt % were considered to negligible and the detected monomers to arise from cell membrane components. Indeed no PHA could be extracted [39,40] from the biomass of *P. putida *KT2440 cultivated on citrate with a C/N ratio of 15 g g^-1 ^although 5.1 wt % "PHA" were detected by GC (data not shown).

### Calculations

#### Specific uptake and production rates

The specific gluconate uptake rate and the specific 2-ketogluconate production rate during the exponential phase were calculated based on the PHA-free biomass. All the calculations involving gluconate and 2-ketogluconate were done in g C to facilitate comparison with the TOC values.(1)(2)

The specific carbon consumption rate q_C* _was defined as the rate at which carbon is consumed by the cells for respiration and production of biomass. Assuming that there is no accumulation of gluconate in the periplasm, q_C* _can be deduced from the specific gluconate uptake rate and the specific 2-ketogluconate production rates. Before gluconate depletion:(3)

and afterwards:(4)

Similarly, the specific nitrogen uptake rate was defined as:(5)

For the PHA accumulation phase other equations must be applied since the cells do not grow anymore at μ_max_.(6)

This equation implies that the PHA-free biomass (X_r_) stays constant which was the case for *P. putida *KT2440 but not for *P. putida *KT2442. Therefore the average between the two extremes values was considered for the latter strain.

The specific PHA production rate was calculated with the same equation.(7)

#### Growth and PHA yields

The growth yields for carbon and nitrogen were calculated at the end of the exponential phase using the PHA-free biomass values.(8)(9)

The PHA yield for carbon at the end of the cultivation was calculated in the following way:(10)

#### Respiratory quotient

The respiratory quotient (RQ) is defined as the ratio between the carbon production rate (CPR) and the oxygen uptake rate (OUR).(11)(12)(13)

The gas flow rate leaving the reactor is linked with the gas flow entering by the following equation.(14)

## Abbreviations

C(Gln): gluconate-based carbon concentration [g L^-1^]; C(2-KGln): 2-ketogluconate-based carbon concentration [g L^-1^]; CDW: cell dry weight [g L^-1^]; CPR: carbon dioxide production rate [mol L^-1 ^h^-1^]; F_G_: gas flow [L h^-1^]; Gln: gluconate; 2-KGln: 2-ketogluconate; μ_max_: maximum specific growth rate [h^-1^]; NH_4_-N: ammonium nitrogen [g L^-1^]; OD_600_: optical density (of a cell culture) at 600 nm [-]; OUR: oxygen uptake rate [mol L^-1 ^h^-1^]; mcl-PHA: medium-chain-length polyhydroxyalkanoate; scl-PHA: short-chain-length polyhydroxyalkanoate; q: specific uptake/production rate [g g^-1 ^h^-1^]; TOC: total organic carbon [g L^-1^]; RQ: respiratory quotient [mol mol^-1^]; V_m_: molar volume [L mol^-1^]; V_w_: working volume [L]; X_r_: PHA-free biomass [g L^-1^]; y: molar fraction; Y_Xr/C_: growth yield for carbon [g g^-1^]; Y_Xr/N_: growth yield for nitrogen [g g^-1^]; Y_PHA/C_: PHA yield for carbon [g g^-1^].

### Indexes

0: initial; C*: carbon effectively consumed by the cells for respiration and: biomass production; C(Gln): carbon present in gluconate; C(2-KGln): carbon present in 2-ketogluconate; e: end of exponential phase; f: final; in: inlet gas to the bioreactor; out: outlet gas from the bioreactor.

## Competing interests

The authors declare that they have no competing interests.

## Authors' contributions

SF performed the laboratory experiments and drafted the manuscript. MZ advised the experimental design and together with SP revised the manuscript. All authors read and approved the final manuscript.
